# Findings of an evaluation of a sexual and reproductive health programme in a humanitarian setting for the forcibly displaced Myanmar nationals in Cox’s Bazar, Bangladesh

**DOI:** 10.7189/jogh.14.04146

**Published:** 2024-09-06

**Authors:** Meftah Uddin Mahmud, Shaki Aktar, Shakil Ahmed, Tanjeena Tahrin Islam, Dipika Paul, Sayed Rubayet, Fauzia Akhter Huda

**Affiliations:** 1 Maternal and Child Health Division, icddr,b, Dhaka, Bangladesh; 2 Department of Obstetrics and Gynecology, McMaster University, Hamilton, Ontario, Canada; 3 Department of Data Science, RMIT University, Melbourne, Australia; 4 Ipas Bangladesh, Dhaka, Bangladesh

## Abstract

**Background:**

Since August 2017, around 940 000 forcibly displaced Myanmar nationals (FDMN), mostly women and children, have fled persecution in Myanmar and arrived in the refugee camps across the border in Cox’s Bazar, Bangladesh. This large-scale humanitarian crisis created an urgency for sexual and reproductive healthcare-related services among many of the sexually assaulted FDMN women and girls. Ipas, an international non-governmental organisation (NGO) that has been working on expanding access to safe menstrual regulation, post-abortion care, and family planning services in Bangladesh since 2011, initiated an emergency humanitarian response programme in the refugee camps in Cox’s Bazar in 2017 for the victim FDMN women and girls who were in desperate need of care. To understand the implementation process and the scope of sustainability and scale-up of Ipas’s programme in the current humanitarian settings, icddr,b, a Bangladesh-based international health research institution, conducted an evaluation study.

**Methods:**

Due to the emergency crisis situation, Ipas could not collect baseline data while initiating its humanitarian response programme in 2017. Only a post-evaluation was carried out by icddr,b from August to December 2022 based on a desk review, health facility observation and assessment, qualitative interviews, and a stakeholder consultation workshop.

**Results:**

In collaboration with relevant stakeholders from the Government of Bangladesh and local and international NGOs, Ipas performed structural renovation and logistical arrangements to ensure facility readiness within the camps. Until December 2022, it provided comprehensive training on menstrual regulation, post-abortion care, and family planning services to around 700 service providers from partner organisations and expanded its activities from 8 to 51 service delivery points in 23 camps. Overall, 42 213 FDMN women received menstrual regulation and post-abortion care, while 339 334 received family planning services from these facilities, with a growing trend over time.

**Conclusions:**

Despite the challenges and barriers inherent to a humanitarian setting, Ipas’s programme activities have achieved significant progress in providing menstrual regulation, post-abortion care, family planning services, and trauma/survival-centred care to the FDMN women and girls. A flexible approach, stakeholder coordination and commitment, cohesive methods for health systems strengthening, and community engagement were instrumental to the success of Ipas’s humanitarian response programme.

Worldwide, nearly one-third of an estimated 210 million pregnancies occurring each year are unplanned, with some resulting in unwanted births and others being terminated through induced abortions [1,2]. In countries with restrictive abortion laws or with inadequate or poor-quality abortion services, women turn to unqualified providers, risking serious health issues or death [3]. Despite being an avoidable cause of maternal mortality and morbidity and a priority issue for public health [4], almost 47 000 women died from unsafe abortions in 2008, and nearly five million of the survivors had long-term health consequences [5,6].

Ipas is one of the few international non-governmental organisations (NGOs) working globally to advance reproductive justice; since its founding in 1973, its mission has been to establish a robust ecosystem for safe abortion and contraception services [7]. The NGO currently operates in 22 countries across the world and executes its policies through strategic collaboration with local and national NGOs [8]. Ipas started its journey in Bangladesh in 2011 to reduce unsafe abortion-related deaths and damage by enhancing post-abortion care (PAC) that included menstrual regulation (MR), management of abortion-related complications, and family planning (FP) services [8].

In August 2017, around one million forcibly displaced Myanmar nationals (FDMN), also known as the Rohingyas, fled persecution from the Rakhine state of Myanmar to avoid being killed and expelled by the state forces and took shelter across the border in Cox’s Bazar, Bangladesh, the largest and most densely populated refugee camp in the world [9,10]. During this humanitarian crisis, a significant number of FDMN women and girls were subjected to sexual assault and were in dire need of medical attention [11]. This created an urgent need for the availability and accessibility of sexual and reproductive healthcare services in the camps [12].

In coordination and collaboration with relevant stakeholders from the Government of Bangladesh, United Nations (UN) agencies, and local and international NGOs, Ipas Bangladesh initiated an emergency humanitarian response programme in 2017 to provide comprehensive MR/PAC/FP services, and trauma/survival-centred care to the affected FDMN women and girls in the camps [13].

icddr,b, an international health research institute based in Dhaka, Bangladesh, carried out an evaluation of the activities implemented through Ipas’s humanitarian response programme, their success in achieving the targeted outcomes, the programme’s commitment to ensure quality service delivery, and the appropriateness of its utilisation of resources. It also sought to identify any implementation bottlenecks the programme may have encountered. The findings of the evaluation may help to justify the sustainability and scaling up of Ipas’s existing MR/PAC/FP services and trauma/survival-centred care in the current humanitarian settings in Cox’s Bazar, Bangladesh, while the programme design could be replicated and recommendations from this evaluation can be adopted by other organisations and/or countries working in humanitarian settings.

## METHODS

The humanitarian response programme was implemented in the Ukhia and Teknaf sub-districts of Cox's Bazar, Bangladesh. Due to the ongoing emergency, Ipas could not collect any baseline individual-level information at the start of the programme. Therefore, the icddr,b evaluation team could only conduct a post-evaluation of the programme from August to December 2022, through a desk review, health facility observation and assessment, qualitative interviews, and a stakeholder consultation workshop.

### Desk review

The research team reviewed all available training manuals and guidelines relevant to MR/PAC/FP services, as well as various programme reports from Ipas, to understand their programme activities and service utilisation (Table S1 and S2 in the **Online Supplementary Document**). Through desk review methods, we extracted quantitative data on service utilisation and information on background history of the programme.

### Health facility observation and assessment

Through this approach, we aimed to collect quantitative data on facility readiness. Specifically, we compiled a list of healthcare facilities supported by Ipas in the refugee camps in Cox’s Bazar to establish a sampling framework. Based on random selection from this framework, we visited several Ipas-supported health facilities to observe their activities and collect essential information regarding their readiness to provide MR/PAC/FP services and trauma/survival-centred care. We used a structured checklist adapted from the validated tool of the Bangladesh Health Facility Survey 2017 [15] data collection. We also reviewed health facility records, including registers and referral books, to collect data on MR/PAC/FP/trauma/survival-centred care service use by FDMN women and girls in the previous years. Additionally, the evaluation team engaged with some beneficiaries on-site to understand their satisfaction with or adherence to the services they had received.

### Qualitative interviews

We conducted in-depth interviews with service providers, held focus group discussions with program managers, and carried out key informant interviews with collaborating and implementing partners of Ipas and facility managers. These interviews explored the various aspects of the service delivery process, the pathway for providing sexual and reproductive health (SRHR) services, quality assurance measures, supply chain management, challenges, opportunities, accomplishments, and programme adherence. During our observation and assessment of health facilities, we approached the service providers offering MR/PAC/FP services for in-depth interviews, which we conducted with their agreement. We also reached out to Ipas’s programme managers to organise focus group discussions. With their assistance, we identified key stakeholders associated with Ipas Bangladesh’s humanitarian response programme, whom we then contacted through a snowballing approach to organise key informant interviews.

### Stakeholder consultation workshop

We organised a stakeholder consultation workshop with the relevant key stakeholders from the Government of Bangladesh, NGOs, collaborating and implementing partners, and Ipas officials from the local and national levels to share and validate the programme evaluation findings, as well as to draw recommendations and determine a way forward for the programme.

### Data analysis

We performed descriptive analyses on the health facility observation and assessment data using Stata, version 15.0 (StataCorp LLC, College Station, Texas, USA). All qualitative interviews were digitally recorded and transcribed. The qualitative analysis process involved synthesising and interpreting findings to provide explanations, as well as comparing and contrasting findings both within and across different groups of respondents. We then extracted specific statements from each transcript for presentation in our manuscript. We also analysed quantitative data extracted during the desk review to generate evidence of the activities conducted by Ipas Bangladesh, while qualitative analysis was undertaken to comprehend the procedural aspects of these activities.

### Ethical consideration

The Ethical Review Committee of icddr,b conducted an ethics review and approved our study, ensuring adherence to ethical standards in research involving human subjects. We took stringent measures to protect the privacy and rights of Rohingya individuals and collected no personal data. We otherwise maintained the anonymity and confidentiality of all collected data, with access restricted to authorised personnel only, including designated study team members, ensuring adherence to ethical guidelines and data integrity. The participants of the interviews and focus groups provided written informed consent for participation and recording.

## RESULTS

### Desk review

**Figure 1:**
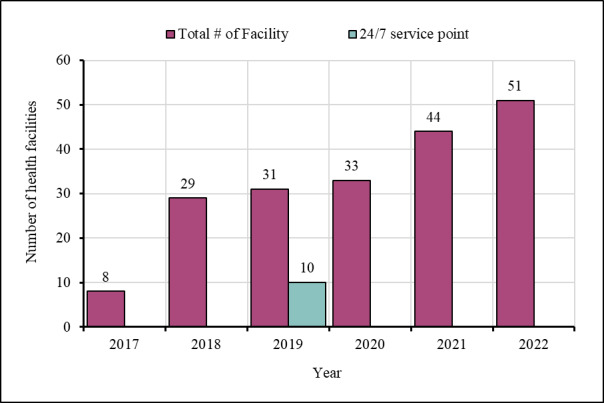
Number of Ipas supported health facilities in Cox’s Bazar.

Ipas, in collaboration with the United Nations Population Fund (UNFPA), conducted an assessment in 2017 to determine whether the existing health facilities located in the camps were offering MR/PAC/FP services. The findings showed that FDMN women with unwanted pregnancies were being returned from the health facilities due to the unavailability of comprehensive MR/PAC/FP services. Therefore, Ipas communicated with potential donors, including the Packard Foundation, the Global Fund, the Government of Canada, and the UNFPA for fundraising. Through UNFPA, Ipas advocated for integration of MR/PAC/FP services in the existing programmes of some of the local and international NGOs who were providing other health services in the camps by that time [14]. In collaboration with relevant stakeholders from the Government of Bangladesh and local and international NGOs, Ipas performed some structural renovation and logistical arrangement in eight facilities within the camps to ensure facility readiness and started providing MR/PAC/FP services. By December 2022, Ipas expanded their activities to 51 service delivery points located in 23 camps (**Figure 1**).

**Figure 2:**
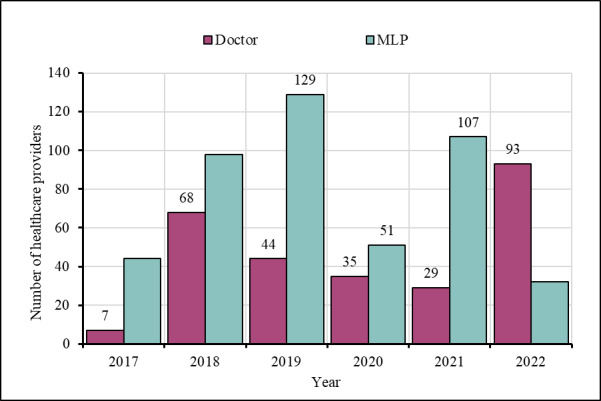
Number of health care providers received basic and refresher training (2017–22).

Since 2017, the Reproductive Health Services Training and Education Program (RHSTEP) and the Bangladesh Association for Prevention of Septic Abortion (BAPSA), two of the Ipas’ major implementing partners, have been providing training to different cadres of service providers at their facilities on MR/PAC/FP services, counselling and awareness raising (**Figure 2**). Following World Health Organization (WHO) and Government of Bangladesh guidelines, separate training modules were designed for doctors, mid-level providers (nurses, midwives, family welfare visitors, and paramedics), community health workers (CHWs), and volunteers. Ipas also arranged regular refresher training for service providers to ensure quality.

**Figure 3:**
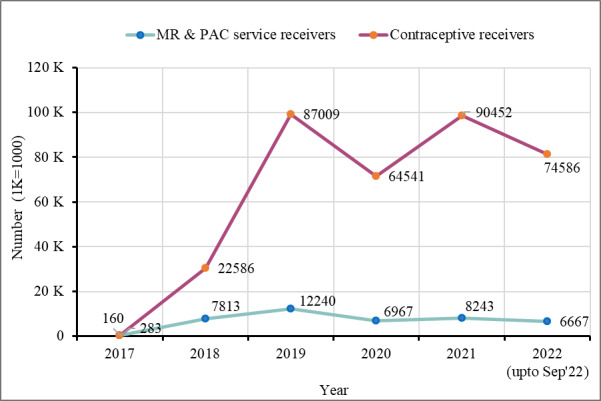
Utilisation of MR/PAC/FP services in health facilities in the camps.

Ipas offered its services through three categories of health facilities in the camps run by different collaborating partners. The field hospitals are the top-level facilities among those where all types of services including counselling to operative procedures were provided; health posts, the lowest-level service centres, provided non-invasive services only (**Table 1**). From these facilities, about 339 334 Rohingya women received contraceptive services, while 42 213 received MR/PAC services between August 2017 and September 2022, with an increase in service utilisation over time (**Figure 3**).

**Table 1:** Types of health facilities and service availability by Ipas

Types of health facilities	Services availability
Health post	Counselling on all types of SRHR services; short-acting contraceptive methods; PPFP methods; PAFP methods; MRM; trauma/survival-centred care; psychological support
Primary health care centre	Counselling on all types of SRHR services; short and long-acting contraceptive methods; PPFP methods; PAFP methods; MRM; MVA; non-complicated PAC services; trauma/survival-centred care; psychological support; CMR
Field hospital (referral point for the health post and primary health care centre)	Counselling on all types of SRHR services; short and long-acting contraceptive methods; PPFP methods; PAFP methods; MRM; MVA; non-complicated and complicated PAC services; trauma/survival-centred care; psychological support; CMR

CMR – clinical management of rape, MRM – menstrual regulation with medication, MVA – manual vacuum aspiration, PAC – post-abortion care, PAFP – post-abortion family planning, PPFP – post-partum family planning, SRHR – sexual and reproductive health programme

**Figure 4:**
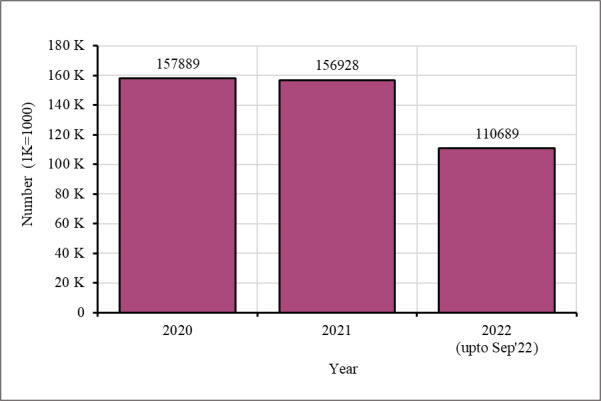
Number of beneficiaries received counselling services and awareness sessions through Ipas’s SBCC activity.

Ipas continuously aimed to improve community sensitisation, counselling, and awareness-raising activities to increase service uptake. It deployed social and behaviour change communication (SBCC) officers in each camp to supervise the activities of CHWs who delivered messages on FP to the Rohingya women during their routine household visits (**Figure 4**).

### Health facility observation and assessment

**Figure 5:**
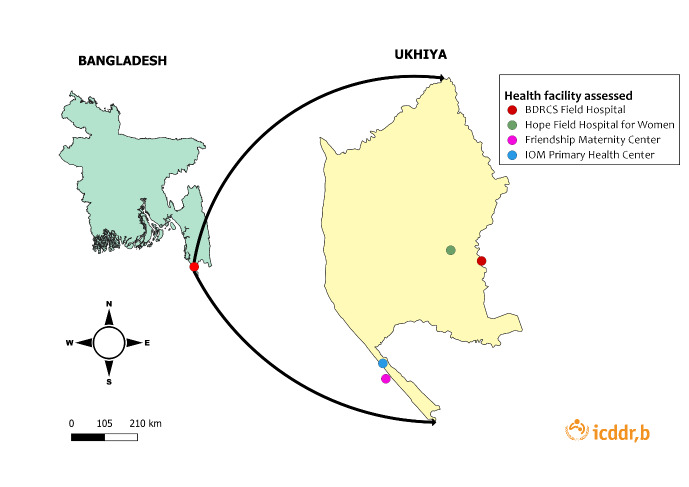
Map of the health facilities observed and assessed in this study.

The evaluation team physically visited four Ipas-supported health facilities in the camps founded by the International Organization for Migration (IOM), the Hope Foundation, Friendship, and Bangladesh Red Crescent Society for observation and assessment (**Figure 5**).

All of the facilities were well-structured, without significant damage to their walls, doors, and ceilings. General cleanliness was satisfactory, with 95% positive responses; patients’ waiting areas and facility floors were clean and protected from sunlight and rain. However, few hand washing stations had visible dirt or waste and some did not have soap. On average, 88% of the lavatory facilities were appropriately placed in the service centres with privacy, functionality, and cleanliness. Separate toilets for men and women were available in all the service centres. All facilities had adequate and clean water supply. The water supply systems were properly functioning and have not gone through any severe shortages in the past years. However, the power supply was interrupted several times, though the facilities reportedly had at least one functioning generator and one assigned generator operator. Overall, 96% of all the quality assurance and supervision activities were routinely carried out across the facilities (Figure S1 in the **Online Supplementary Document**).

Moreover, comprehensive FP services, including short- and long-acting modern contraceptive methods and MR with medication and manual vacuum aspiration services were available in the facilities. The service providers were well trained, skilled, and provided services (Table S3 in the **Online Supplementary Document**) per their specific guidelines.

All necessary commodities and medicines were available in the facilities sufficiently, stored in clean and well-ventilated rooms, and organised according to their date of expiry while maintaining 20 days’ buffer stock. However, in one out of the four facilities, medicines were not organised according to the expiry date (**Table 2**). The service providers ensured privacy of the patients and used sterile instruments during providing manual vacuum aspiration services. Medical wastes were disposed into the designated colour-coded bins and taken to the waste disposal area before two-thirds of the bins were full. Out of the four facilities visited, the incinerator was out of order in one facility, so its waste was sent to another nearby health facility with a functioning incinerator.

**Table 2:** Storage of medicines and commodities in the health facilities visited

Characteristics	Frequency (n = 4)
Availability of storage for medicine and commodities	
*Yes*	4
*No*	0
Medicines and commodities were organised separately	
*Yes*	4
*No*	0
Medicines were organised following expiry date	
*Yes*	3
*No*	1

During health facility visits, the evaluation team talked with some of the beneficiaries who came to receive different services. Almost all of the women reported that the providers were welcoming and respectful to them, and they were satisfied with the services they received. The women who came for family planning services mentioned that the service providers explained all the available methods and allowed them to choose one voluntarily. The MR/PAC service beneficiaries also expressed their satisfaction regarding the procedures they had received and mentioned that the providers had performed the services after obtaining informed consent and maintaining privacy. Almost all of the women were satisfied with the service quality and stated that if required, they would visit the facilities again and recommend their relatives and friends to take services from those facilities.

### Qualitative interviews

To understand the service delivery process, challenges, accomplishments, and compliance with the humanitarian response program of Ipas, we conducted 17 interviews, including five in-depth interviews with service providers, 12 key informant interviews with collaborating and implementing partners and the facility managers, and a focus group discussion with Ipas staff. We identified the respondents for the interviews through consultation with the programme managers of the relevant field.

All the service providers mentioned providing a wide range of MR/PAC/FP-related services to the Rohingya women and girls. One of them stated:

Basically, I work here as an MR/PAC/FP service provider. At first, I counsel my clients, talk about contraceptive methods and related side effects with them [the clients], and then provide services.

Findings from the in-depth interviews revealed that to ensure service quality, Ipas, in collaboration with other partner organisations, delivered regular training for the service providers to strengthen their capacity. The training covered the services provided, as well as community sensitisation and mobilisation. In collaboration with RHSTEP and BAPSA, Ipas provided training to over 700 service providers including doctors, nurses, midwives, paramedics and CHWs. A service provider mentioned that:

I joined here in June 2022… prior to starting my work, I received a training of 14 days from BAPSA & Ipas. They taught us the theory and then gave us practical training on MR/PAC/FP… yes, I also received two days’ refresher training in September [2022].

In addition to the training, Ipas provided logistics and equipment support to their partner organizations to facilitate program implementation. A participant of the focus group discussion stated that:

Our main components are providing logistics, equipment including technical supports to partner organizations to provide quality MR/PAC/FP services…

We found that Ipas had initiated a robust SBCC activity to uproot taboos about community perception regarding MR/PAC/FP services. It used an interactive ‘values clarification for action and transformation’ tool to reduce stigma for the Rohingya community. Ipas also involved the local leaders and imams (religious leaders) in the training sessions to maximise community engagement. One key informant mentioned that:

We found that the Rohingya imams are very powerful in their community. So, we involved Cox’s Bazaar Islamic Foundation, and with their help, we trained the Bangladeshi imams first… the Bangladeshi imams then provided training to the Rohingya Imams. Now, Rohingya Imams are disseminating MR/PAC/FP-related messages…

Ipas’s proactive approach to advocacy, coordination, and policy-level communication has also been a key mediator to the successful implementation of the programme, as identified by some of the key informants. Ipas fosters strong collaboration with the local and central Government of Bangladesh, UN agencies, and national and international NGOs. As a result, long-acting reversible contraceptives (LARC) were introduced in the Rohingya camps. One of the key informants stated that:

We continued our advocacy with the DGFP [Directorate General of Family Planning] for LARC services. The RRRC [Refugee Relief and Repatriation Commissioner] and UNHCR [United Nations High Commissioner for Refugees] jointly conducted an assessment and reported that their [Rohingya population’s] family size was increasing in such a way that this population might get doubled in a year… that report helped in our advocacy process… and the Government of Bangladesh finally took the decision of providing LARC to the eligible Rohingya women.

Participants from the key informant interviews considered the services provided by Ipas to the Rohingya community to be significant, relevant, and efficient in the current context, while broadly acknowledging their strength in implementing SRHR programmes in humanitarian settings. One of them mentioned:

The Ipas people are very ‘professional’… they are always ready to support us. They do a lot of work in the area of family planning and reproductive health. They also give lots of feedback, and the kind of feedback they provide is so practical… I mean… those have some justifications… so, in a nutshell, Ipas’s good communication, professional work, and good relationship with the government, RRRC, Civil Surgeon, and all the partners here, including the CIC's [camp in charge] in the camps helped in making the program successful.

### Stakeholder consultation workshop

The evaluation team arranged a stakeholder consultation workshop, attended by 21 participants from 14 different organisations involved in the Rohingya response, such as government and UN agencies, and local, national, and international NGOs. The aim of the workshop was to share the preliminary findings from the evaluation and formulate recommendations in discussion with the stakeholders on the scope of sustainability and scale-up of the programme in humanitarian contexts.

Initially, the study team presented the preliminary findings of this evaluation to key stakeholders, including all the results from the desk reviews, health facility observations and assessments, and qualitative interviews. Following the presentation, stakeholders engaged in an open discussion on the importance of sustainability and scale-up of the programme. Subsequently, the participants were divided into two groups: one group summarised their recommendations on the long-term sustainability of the programme, and the other group summarised their recommendations on the opportunity for scaling up the programme.

Based on the preliminary findings shared in the workshop and group work activity, the key stakeholders identified six major areas through which the programme could be made sustainable and scaled up. The areas included advocacy with the government; community engagement; engaging more partner organisations; resource mobilisation; needs assessment in the remaining refugee camps where MR/PAC/FP services are not available; and capacity building.

When discussing sustainability, stakeholders believed it could not be achieved without the participation of the government. They suggested that Ipas can enhance its advocacy efforts with the Government of Bangladesh to increase funding. Additionally, effective inter-ministerial coordination, such as coordination between the Ministry of Health and Family Welfare, the Ministry of Disaster Management and Relief, and the Ministry of Religious Affairs, was also seen as essential to sustaining the programme.

The stakeholders similarly mentioned that a funding crisis could be the biggest obstacle to sustainability. Therefore, they suggest that Ipas consider advocating to partner organisations for their commitment to cost-sharing for MR/PAC/FP services. Moreover, Ipas could consider expanding their partnerships with other organisations. In addition, advocacy for financial support from the Government of Bangladesh, foreign aid, and donor agencies were seen as necessary for sustaining Ipas’s activities.

## DISCUSSION

Monitoring and evaluation are a prerequisite to realising the success of a program implemented, its accomplishments, and to identify the bottlenecks to its activities [16]. It is also important to recognise if the commitment of the programme to ensure and demonstrate quality service delivery, the appropriate use of resources, and the highest transparency were adequately maintained. We conducted our evaluation of Ipas’s humanitarian response programme to assess its efficiency and quality, and to provide recommendations on its sustainability and scale-up in the humanitarian setting in Cox’s Bazar, Bangladesh.

The Government of Bangladesh, UN agencies, and other local and international NGOs prioritised emergency obstetric care service provision to pregnant women residing in the refugee camps, as in any other global humanitarian settlements. A systematic review evaluating the effectiveness of different SRHR interventions in humanitarian crises identified that one out of twenty-six studies focussed particularly on abortion services [16]. Ipas was the first organisation in Bangladesh to implement comprehensive MR/PAC/FP services for the Rohingya women and girls in the camps. These services have the potential to save their lives from adverse health consequences of unsafe abortions and related complications, as well as unintended pregnancies. However, due to a lack of baseline information on the number of children per woman, unmet need for FP, and proportion of complications following unsafe abortions, the evaluation team could not estimate the actual rate of total fertility declines and unsafe abortion-related morbidities and mortalities. Therefore, we used a post-evaluation approach similar to that adopted in assessing several SRHR programmes in other humanitarian settings globally [18–22]. Our evaluation found that Ipas has been making significant contributions to increasing the accessibility and availability of high-quality MR/PAC/FP services at the healthcare facilities located inside and around the camps.

In times of emergency and in humanitarian settings, incidents of sexual assault and other forms of violence against women are more prevalent, while SRHR services provide minimal standards of health care without maintaining any human rights [23,24]. Despite significant difficulties and obstacles within the camps, Ipas has progressively improved and expanded the coverage of its SRHR services in Ukhiya, Teknaf, and Bhasan Char over time. This was only possible due to their dynamic nature, professional indulgence, and strong collaboration with relevant stakeholders and some committed, cooperative, and motivated partner organisations. From initially preparing the health facilities to deliver the services, Ipas has shown a high level of efficiency, productivity, competency, and knowledge at every stage of the humanitarian crises to make SRHR services easily available and accessible to the FDMN community. We recognised Ipas’s commendable efforts in designing the programme with careful consideration for gender and cultural sensitivity. Ipas made the strategic decision to recruit exclusively female service providers, enhancing the acceptability of services among Rohingya women, while also actively engaging men and influential figures within the community, including local and religious leaders. This inclusive approach ensured that the programme was culturally relevant and acceptable to the Rohingya community, both in its design and implementation.

We observed that Ipas successfully continued its humanitarian response program in selected health facilities, with a particular focus on service availability, adequate infection prevention strategies, and supply chain and waste management. This reflects Ipas’s good governance, proper monitoring, quality assurance, and dedication to the program. To strengthen the service providers’ capacity and to ensure high-quality services, Ipas delivers provider-specific strategies and arranges regular training/refresher training for the providers with on-site mentoring. A special emphasis was placed on counselling on misconceptions, traditional thoughts and beliefs, and superstitions around family planning, so that the service providers can help the Rohingya women and girls understand the pros and cons of each method and assist them in choosing the suitable one. Consequently, we observed high satisfaction among the service recipients, indicating the success of Ipas's SRHR programme in the humanitarian context of Bangladesh. Without Ipas’s assistance, it would be difficult for the Government of Bangladesh and other partner organisations to maintain quality MR/PAC/FP services in the Rohingya refugee camps.

The humanitarian response program of Ipas has some limitations. Out of total 200 health facilities located in 34 Rohingya camps, Ipas is providing their support only in 51 health facilities in 23 camps. Despite their improvement in service coverage over time, further development in this area is essential, as a larger proportion of the Rohingya community is still deprived of these lifesaving services.

### Challenges and barriers

Ipas encountered several challenges and barriers when implementing their programme in the humanitarian setting, with some starting as early as from its inception. However, they took proactive initiatives to address these challenges, as presented below.

#### Language barrier

Language initially posed a significant barrier, leaving many Rohingya women and girls unable to access critical information and services. To overcome this barrier, Ipas developed audiovisual materials, illustrated brochures, and leaflets in the Rohingya language. Leveraging the substantial similarity between Rohingya and the local language of Cox’s Bazar, Ipas recruited providers from the local community, further minimising language barriers. Over time, both service providers and Rohingya community members learned each other’s languages, further alleviating communication challenges.

#### Inclusion of MR/PAC services in minimum initial service packages

The minimum initial service package (MISP) is defined as ‘the minimum, life-saving sexual and reproductive health needs that humanitarians must address at [the] onset of an emergency’ [25]. At the onset of the Rohingya crisis, MR/PAC services were not included in the MISP, leading to their absence in most health facilities’ service packages. Recognising the urgent need for these services amid reports of mass rape, Ipas advocated with the UNFPA, ultimately resulting in the global adoption of MR/PAC service in MISP.

#### Initiation of the LARC service

Delays in launching the LARC services for the Rohingya community stemmed from the lack of government and other stakeholder approvals. Persistent advocacy efforts by Ipas towards government and UN agencies eventually secured approval for LARC services after nearly a year of advocacy.

#### Providing training at the initial stage

With only eight facilities initially, providers were overburdened with high service expectations, leading to extended waiting times for clients. To address this, Ipas provided on-site training to providers by sending trainers to each facility instead of arranging separate formal training sessions.

#### Stigma and religious taboo around family planning services

Establishing family planning initiatives among the conservative Rohingya community was challenging due to their deeply rooted cultural and religious beliefs. Given the predominant leadership of men within the community, Rohingya women often hesitated to seek family planning methods, constrained by their limited decision-making autonomy. This contributed to a rise in unintended pregnancies, many of which led to unsafe abortions. In response, Ipas launched SBCC activities such as ‘Learning by Playing’, tailored for both men and women in the community to raise awareness about MR/PAC/FP services. Additionally, engaging religious and social leaders proved instrumental in reducing the stigma and taboo associated with these services.

#### Community mobilisation at the early stage

Given the transient nature of the community post-influx, Ipas focussed on reaching all health facilities surrounding Rohingya camps with information about their supported service centres, thereby facilitating referrals for MR/PAC/FP services.

#### Inadequate number of health facilities and providers

Ipas’s SRHR services were initially available in only eight facilities, after which it expanded its services gradually to 43 more facilities to address long waiting times and privacy concerns.

#### Providers’ stigma

To address providers’ tendency to impose personal preferences on clients regarding contraceptive method choice and their stigma regarding MR/PAC, Ipas initiated ‘whole-site orientation’ activities, sensitising all facility members and clarifying values using ‘values clarification for action and transformation’ tools.

#### Quality of care

Maintaining service quality in the emergency humanitarian setting was challenging due to the frequent turnover of skilled providers, resource constraints, and limited space. Ipas addressed this by increasing the number of facilities and skilled providers through effective communication, collaboration, training, and advocacy.

#### Fund constraints

Shortage of funding resulted in turnover of trained staff, posing a significant challenge to programme continuity for Ipas.

#### Sustainability

The transition and phase-out of the project raise questions about the sustainability of Ipas’s efforts by the Government of Bangladesh, posing a key challenge for the organisation.

### Recommendations

We found sufficient evidence to deem Ipas’s humanitarian response programme successful, underscoring the importance of prioritising the sustainability of these efforts. Simultaneously, the programme awaits expansion into other refugee camps, marking a crucial phase in its future strategy. However, several critical issues require attention in these new phases:

1. Conducting a comprehensive needs assessment, including health facility readiness, workforce assessment, and logistical supply, in the remaining camps where Ipas’s programme activities have yet to be introduced.
2. Identifying barriers and challenges related to low utilisation of LARC and segmenting couples according to their needs to provide timely and accurate information, thereby increasing utilisation rates.
3. Initiating effective advocacy with the Government of Bangladesh to introduce permanent methods of family planning among eligible Rohingya couples in the camps.
4. Establishing community support groups or recruiting volunteers to strengthen community mobilisation efforts and fostering strong linkages with CHWs to dispel superstitions surrounding contraceptive methods, with a particular focus on LARC and permanent methods.
5. Developing a comprehensive SBCC strategy on SRHR services and arranging robust awareness campaigns through easily understandable visual materials, leaflets, brochures, posters, and demonstrative billboards in camp areas.
6. Identifying and implementing innovative approaches to involve men in family planning service delivery, counselling, and awareness campaigns.
7. Ensuring quality of care through regular performance-based reviews of service providers and devising strategies to mitigate trained staff turnover.
8. Motivating current partners and donor organisations to allocate resources toward supporting MR/PAC/FP service provision, and exploring avenues to develop new partnerships. Advocacy efforts for financial support from the Government of Bangladesh, foreign aid, and donor organisations should persist.

### Study limitations

We encountered several limitations during this evaluation. First, due to the urgent nature of the humanitarian crisis, neither Ipas nor any other organisation was able to conduct a population-level baseline assessment to understand the SRHR needs of Rohingya women and girls when the influx began. Consequently, lacking baseline data prevented us from estimating the impact of Ipas's humanitarian response programme in alleviating the SRHR burden of this population. Despite recognising the potential value of alternative methods to estimate baseline conditions, time and resource constraints prevented us from exploring such options, although they could have strengthened the evaluation’s robustness. Second, given the complex and fluid nature of crises in humanitarian contexts, no predefined metrics were established to evaluate the success of SRHR programmes in other settings like Syria and Iraq [26,27], leading them to consider an increase in service utilisation as indicative of success – a methodology mirrored in this study. Third, we could not accurately estimate the direct impacts of services provided by Ipas, such as reductions in maternal mortality ratio or increases in contraceptive acceptance rates, due to the lack of available data for FDMNs residing in the camps in Cox’s Bazar. Furthermore, our literature review did not identify any papers evaluating SRHR programmes in similar humanitarian settings, thus limiting our ability to compare findings and draw comprehensive recommendations.

## CONCLUSIONS

Considering the scarcity of resources resulted in a critical humanitarian emergency situation, management of SRHR-related issues requires thoughtful and specific service delivery packages. We attempted to identify the key elements that made Ipas’s humanitarian response programme successful to inform sustainability and scale-up in other refugee camps. While conclusions cannot be drawn with the small sample size used in this evaluation, the programme data indicate that, despite the challenges and barriers embedded within a humanitarian setting, the activities of Ipas in response to the Rohingya crisis were promising and have achieved significant increase in utilisation of MR/PAC/FP services and trauma/survival-centred care among the Rohingya women and girls. Ipas’ efforts based on a flexible approach, stakeholder coordination and commitment, cohesive method for health systems strengthening, and community engagement were instrumental in the successful implementation of the programme. Some promising practices, such as advocacy, coordination, and policy-level communication; capacity building of the service providers; and joint supervision by Ipas alongside implementing and collaborating partners significantly contributed to the programme’s improvement.

## Additional material


Online Supplementary Document
