# Supplementary material for: Findings of an evaluation of a sexual and reproductive health programme in a humanitarian setting for the forcibly displaced Myanmar nationals in Cox’s Bazar, Bangladesh
Source: J Glob Health. 2024 Sep 6;14:04146. doi: 10.7189/jogh.14.04146 (PMC11377969; doi:10.7189/jogh.14.04146)
Supplement: Online Supplementary Document [file jogh-14-04146-s001.pdf]

Additional materials
Online Supplementary Document.

Supplementary Figure

Figure S1: Overview of facility observation.

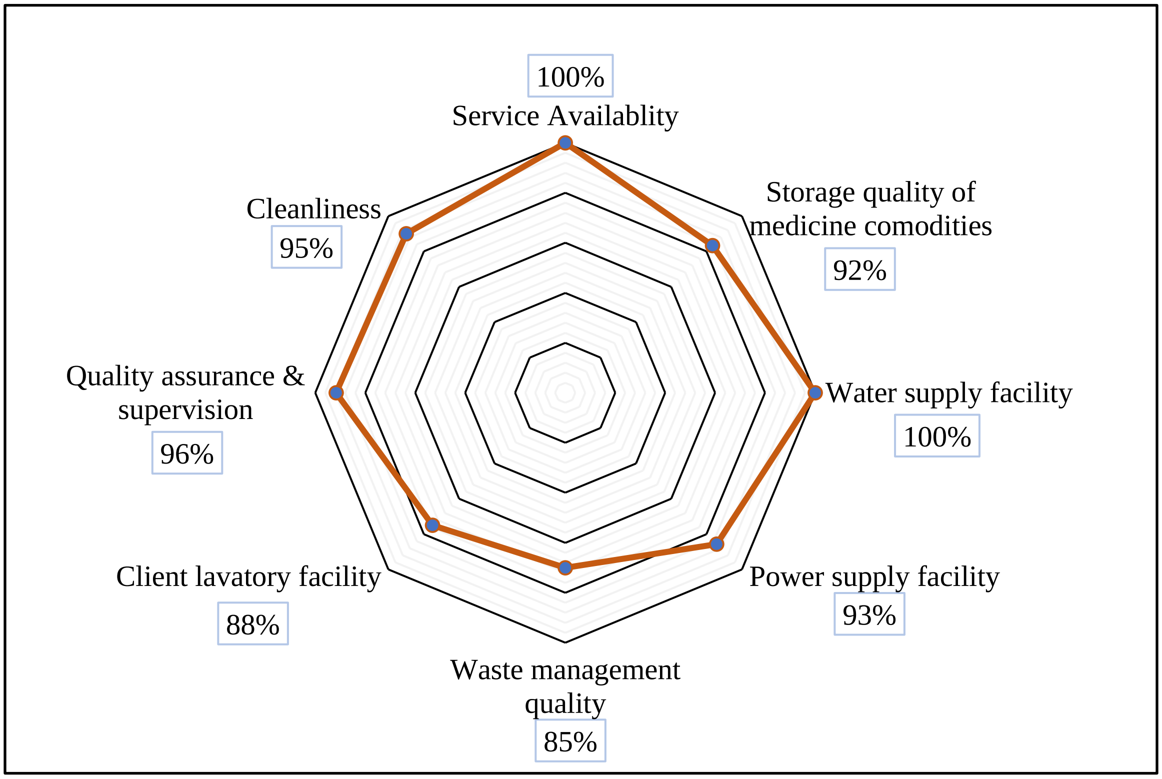

Supplementary Tables

Table S1: Ipas reports reviewed as part of the program evaluation

Sl. No.	Program Reports of Ipas Bangladesh	Reporting Period
1	Emergency Response for Availability and Accessibility of Quality MR, PAC Services for Rohingya Refugees in Bangladesh & Strengthening MR, PAC and FP Services through IPAS Bangladesh	Sep 2017- Jun 2022
2	Sexual and Reproductive Health Program for Rohingya Refugees in Bangladesh	Nov 2017- Mar 2021
3	Population and Reproductive Health Narrative Report	Apr 2019- Mar 2021
4	Sexual and Reproductive Care for Rohingya Refugees in Bangladesh	Aug 2021- May 2022
5	Improving Sexual and Reproductive Health and Rights of Women and Girls in Humanitarian Settings in Bangladesh	Apr 2020- Mar 2022
6	Emergency Response for Availability and Accessibility of Quality MR, PAC Services for Rohingya Refugees in Bangladesh (Indicator based quantitative report)	Sep 2017-Jun 2022

Table S2: Guidelines and manuals of Ipas Bangladesh evaluated during desk review

Sl. No.	Guidelines and Manuals
1	Abortion Attitude Transformation: A values clarification toolkit for humanitarian audience
2	Menstrual Regulation with Medication Study Guide
3	Clinical Protocol on Menstrual Regulation (MR) Post-abortion Care (PAC) Post-abortion & Postpartum Family Planning (PAFP & PPFP)
4	Bangladesh National Comprehensive Menstrual Regulation (MR) and Post-Abortion Care (PAC) Services Guidelines
5	Woman-Centered Menstrual Regulation and Post-abortion Care Services Trainer's Manual
6	Woman-Centered Menstrual Regulation and Post-abortion Care Services Reference Manual
7	Medical Abortion Reference Guide: Induced abortion at or after 13 weeks gestational age ('second trimester')

Table S3: MR/PAC/FP service availability in the health facilities

Characteristics	Frequency (n=4)
**Availability of short-acting contraceptive methods**	
Yes	4
No	0
**Availability of long-acting contraceptive methods**	
Yes	4
No	0
**Days of contraceptive service availability**	
Every day (except holidays)	4
Specific days of the month/week	0
**Availability of contraceptive guidelines/manual**	
Yes	4
No	0
**Availability of MR services**	
Both MVA and MRM	4
Only MVA	0
Only MRM	0
**Days of MR service availability**	
Every day (except holidays)	4
Specific days of the month/week	0
**Availability of MR guideline/manual**	
Yes	4
No	0
**Availability of PAC services**	
Yes	4
No	0
**Days of PAC service availability**	
Every day (except holidays)	4
Specific days of the month/week	0
